# Structural evolution, photoelectron spectra and vibrational properties of anionic GdGe_*n*_^−^ (*n* = 5–18) nanoalloy clusters: a DFT insight†

**DOI:** 10.1039/d2ra04037a

**Published:** 2022-08-10

**Authors:** Zhaofeng Yang, Aziz U. Rehman, Zhenzhu Cao, Jucai Yang

**Affiliations:** School of Chemical Engineering, Inner Mongolia University of Technology, Inner Mongolia Key Laboratory of Theoretical and Computational Chemistry Simulation Hohhot 010051 Peoples Republic of China yangjc@imut.edu.cn; School of Energy and Power Engineering, Inner Mongolia University of Technology Hohhot 010051 Peoples Republic of China

## Abstract

The structural growth of Gd-doped germanium anionic nanoclusters, GdGe_*n*_^−^ (*n* = 5–18), has been explored *via* quantum chemistry calculations using the mPW2PLYP method and an unprejudiced structural searching technique known as ABCluster. The optimized geometries exhibited that when *n* = 10–14, the structural evolution favors the Gd-linked configuration where the Gd atom as a connector bridges two Ge subgroups, while the Gd atom is encapsulated in a closed cage-like Ge frame when *n* = 15–18. The properties like magnetic moment, charge transfer, relative stability, HOMO–LUMO gap, photoelectron spectra, and infrared and Raman spectra have been predicted. The information of these spectra could provide extra approaches to experimentally determine the electronic structures and equilibrium configuration of these compounds. The largest spin magnetic moment of 7 *μ*_B_ is attained *via* half-filled 4f states. The GdGe_16_^−^ nanocluster is determined to be a superatom because its total valence of 75 electrons can be distributed to the orbital sequence of 1S^2^1P^6^(4f^7^)1D^10^1F^14^2S^2^2P^2^1G^18^2P^4^2D^10^, which complies with not only Hund's rule, but also the spherical jellium model. Particularly, its UV-Vis spectra match well with solar energy distribution. Such materials act as nano multifunctional building units potentially used in solar energy converters or ultra-highly sensitive near-infrared photodetectors.

## Abstract 2

The structural evolution, electronic and vibrational properties of GdGe*_n_*^−^ (*n*=5-18) nanoclusters were studied by quantum chemical calculations, which revealed GdGe_16_^−^ nanocluster is a superatom to the orbital of 1*S*^2^1*P*^6^(4*f*^7^)1*D*^10^1*F*^14^2*S*^2^2*P*^2^1*G*^18^2*P*^4^2*D*^10^.

## Graphical abstract

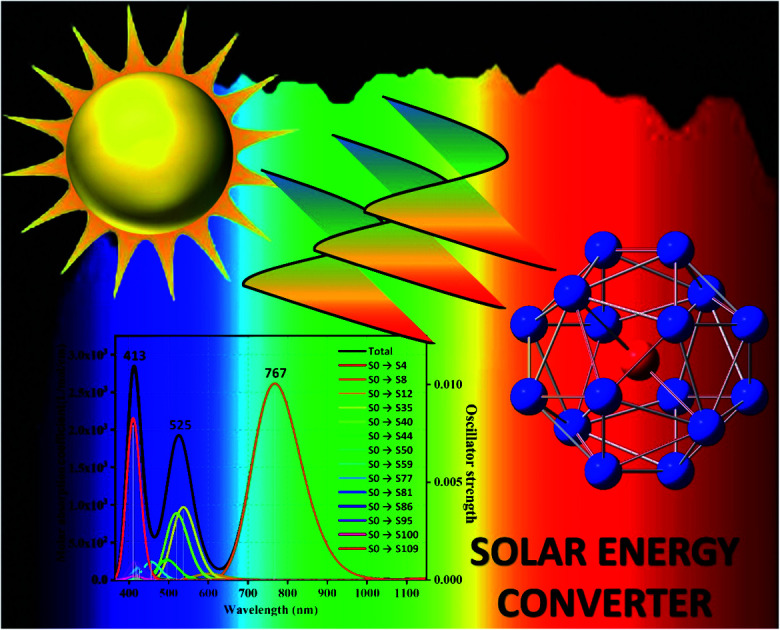

## 1. Introduction

In spite of the fact that silicon has served a critical role in the development of the modern semiconductor industry, it was not the first material which was employed in such gadgets. Indeed, the usage of germanium is well known to build the first transistor.^1,2^ Now people's attention is back to germanium materials due to the fact that germanium-based materials have excellent electron and hole mobilities. Under the premise of low power and high-speed operation, germanium materials are more suitable for electronic equipment than silicon materials.^3,4^ As an alternative to silicon, the use of germanium channel materials in MOS-FET is a strong illustration of its applications.^5,6^ Moreover, germanium has different benefits contrasted with silicon, like higher saturation velocity and lower electronic band gap, which can dispose of the issue of depleting current saturation in MOS-FETs, reduce the operation voltage for the equipment, and improve the performance of photodetector.^7–9^ Germanium-based graphene directly realizes the integration of high-quality graphene and semiconductor substrates, which will promote the wide application of graphene in the semiconductor industry more quickly.^9,10^ On the other hand, exploring the geometric mutations, electronic structures, photoelectron spectra and vibrational modes of nanoalloy clusters have considerable importance due to the fact that nanoalloy clusters play an incredibly essential role in the shift from molecular to condensed matter, with the ongoing progress and widespread application of nanotechnology.^11^

Rare earth metals (REMs) have properties such as high magnetic moments and extremely narrow optical transitions. For example, rare earth molecular crystal has extremely narrow optical transitions and long-lived quantum states, which enables it to be used in fields such as quantum communication and quantum processors, thereby opening up optical quantum systems.^12^ Doping of rare earth metals with Ge clusters not only enriches the properties of germanium-based compounds, but also produces synergistic effects to improve the germanium-based compound's intrinsic properties, thereby obtaining novel functional materials. Ge clusters doped with rare earth metals can be employed as a building block for self-gathered novel functional materials. In addition, the stability of Ge clusters can be improved by doping with rare earth elements since pure Ge clusters possessing only sp^3^-hybridized bonding characteristics are unstable.^13–15^ For instance, ScGe_16_^−^,^16^ LuGe_16_^−^,^11^ and LuGe_17_^+^ (ref. 17 and 18) have been evaluated to be high-symmetry endohedral structures, which give prominence to enhance stability and render them possibly as a building block for new multi-functional nanomaterials. Although REM-doped germanium clusters are not much investigated until now, they are expected to fascinate broader interests since the synergistic effect induced by REM-doped germanium nanoalloys can produce multifunctional nanomaterials with novel properties such as magnetism, photoelectric properties and photosensitivity *etc.*

In terms of experiments, Atobe *et al.* examined the atomic configurations and electronic properties of Ge clusters containing a lanthanide- or transition-atom (MGe_*n*_^−^; M = Lu, Sc, Y, Ti, Zr, Hf, V, Nb, and Ta, *n* = 8–20) *via* scrutinizing the photoelectron spectra (PES) and reactivity.^19^ On the theoretical aspect, Singh *et al.* investigated Th@Ge_*n*_ (*n* = 16, 18, 20) clusters with an *ab initio* calculation, and found that Th-encapsulating improved the stability of Th@Ge_16_ and Th@Ge_20_, besides Th@Ge_16_ has a wide HOMO–LUMO energy gap of 1.72 eV.^20^ Recently, the structural evolution and electronic properties of Lu-doped Ge_*n*_ (*n* = 5–17) compounds in anionic states have been reported.^11^ The 4f orbitals of the Lu atom are fully-filled. Its valence electron configuration is (4f^14^)5d^1^6s^2^. While 4f orbitals of Gd are half-filled, and its electron configuration is (4f^7^)5d^1^6s^2^. To compare the structure and properties of anionic germanium clusters doped with 4f orbital fully-filled Lu atom and 4f orbital half-filled Gd atom, in this study we have conducted a research for seeking the global minimum structure of doping Ge anionic clusters with Gd atom, *i.e.*, GdGe_*n*_^−^ (*n* = 5–18). Global search scheme has been applied to explore their structural features and evolution systematically. Simulation of their PES, infrared and Raman spectroscopy, illumination of the electronic structure and ultraviolet-visible (UV-Vis) spectra of Gd@Ge_16_^−^ as super atom with Frank–Kasper stable configuration has been performed. The findings of this study could help researchers better understand the global minimal structural features and evolution, as well as the stabilities and spectroscopic properties of doping Ge clusters with REM atom, which are highly significant for the construction of electronic equipment, solar cells and so on.

## 2. Computational details

The initial structures search for GdGe_*n*_^−^ (*n* = 5–18) nanoalloy clusters are rooted in two ways: (1) through the ABCluster unbiased global search technique^21–23^ associated with Gaussian 09 package,^24^ more than 400 geometries for each GdGe_*n*_^−^ (*n* = 5–18) nanoalloy clusters were optimized adopting PBE0 scheme^25^ with the pseudopotential ECP28MWB basis set^26^ for Ge atoms and ECP53MWB basis set^27,28^ for Gd atoms. (2) Deduced from the earlier reported structures.^11,17,20^ The low-lying geometries that come from above calculations were re-optimized by using PBE0 combined with cc-pVTZ-PP^29^ and quasi-relativistic *ab initio* effective core potential def2-TZVP^30,31^ basis set for Ge and Gd atoms, respectively. After optimization, vibrational frequency investigations were considered to proof the nature of stationary points. By the above process, mPW2PLYP hybrid functional^32^ were deployed to select isomers for further optimization. However, the mPW2PLYP vibrational frequency was not performed due to limitations of computing capacity. Finally, the single-point energy was done through mPW2PLYP functional with basis set of aug-cc-pVTZ^33^ for Ge and def2-TZVP for Gd.^30,31^ Natural population analyses (NPA) were conducted *via* same scheme. The theoretical PES spectra of these anion nanoalloys were simulated by an outer-valence Green function (OVGF) approximation^34^ combined with aug-cc-pVDZ^33^ and def2-TZVP^30,31^ basis set for Ge and Gd atoms, respectively. The infrared and Raman vibrational spectra of the global minimum structures have been performed by the PBE0 scheme. The DOS (density of states) and PDOS (partial DOS) of GdGe_16_^−^ have been attained by Vienna *Ab initio* Simulation Package (VASP)^35–38^ with PBE-GGA functional.^39^ The projector augmented wave (PAW) was set to explore the inert core electron.^40,41^ To prevent interplay between adjacent nanoalloy clusters, the 40 × 40 × 40 Å edge lengths cubic cells with periodic boundary condition were taken into consideration. The plane wave cut-off energy was set up to 500 eV. The structures, PES spectra, iso-surface maps, and orbitals were created by visualization software of Multiwfn and VMD.^42,43^

Only spin multiplicities of octuplet were reported in this study for GdGe_*n*_^−^ (*n* = 5–18) nano clusters based on the following case. (i) For GdGe_*n*_^−^ (*n* = 1–4) compounds, the spin multiplicities of sextuplet, octuplet, decuplet and twelve states were taken into account. The results revealed that in sextuplet, spin contamination is always present and energies are always high. In twelve state, there are no spin contamination, but energies are also high. Their ground states are either octuplet or decuplet. As can be seen from Fig. S1 in ESI† that GdGe^−^ and GdGe_2_^−^ compounds possess a ^10^∑ and a ^10^B_1_ ground states respectively, which are more stable in energy than that of ^8^∑ and ^8^A′′ excited state by 0.31 eV and 0.36 eV, respectively. For GeGe_3_^−^ alloy, ^8^A_2_ and ^10^Π electronic states compete with each for the ground state since their energy differences are within 0.01 eV. GeGe_4_^−^ compound has ^8^A_1_ ground state, which is more stable than that of ^10^A′′ by 0.65 eV in energy. This situation corresponds to the Ge_*n*_ (*n* = 1–4). The ground states of Ge and Ge_2_ compounds are ^3^P and ^3^∑_g_^−^, respectively. For Ge_3_ compound, ^1^A_1_ (isosceles triangle) and ^3^A_1_′ (equilateral triangle) electronic states compete with each other for the ground state structure.^13^ And the ground state is singlet for pure Ge_4_ clusters.^13,14^ This means that when Gd^−^ anion doped Ge_*n*_ clusters, the 4f electrons of Gd atom do not participate in bonding, and the four valence electrons of Gd^−^ anion interact with the Ge_*n*_ clusters. If the ground states of Ge_*n*_ cluster are originally a triplet, the Gd^−^ anion doped Ge_*n*_ compounds are a decuplet state, and if the Ge_*n*_ clusters are originally a singlet, the Gd^−^ anion doped Ge_*n*_ compounds are an octuplet state. The ground state is singlet for Ge_*n*_ with *n* = 5–18.^13,14^ (ii) Nonetheless, we calculated the energies of the octuplet and decuplet for GdGe_*n*_^−^ (*n* = 5–18) nanoclusters and listed them in Table S1 in ESI,† from which we can see that the energy of decuplet is larger than that of octuplet. Therefore, we only presented octuplet state for GdGe_*n*_^−^ (*n* = 5–18) compounds.

So as to confirm the quality of our employed method, test calculations had formerly been performed through the ROCCSD(T) method for ScSi_*n*_^0/−^ compounds with *n* = 4–9 and compared them with several different DFT functions.^44^ The results proved that only the ground state geometry and vertical detachment energy of ScSi_*n*_^0/−^ compounds calculated by the mPW2PLYP functional agree with that of ROCCSD(T) scheme. Furthermore, the bond lengths of Ge_2_, AgGe, and AuGe compounds calculated *via* mPW2PLYP are 2.38 Å,^45^ 2.45 Å,^45^ and 2.34 Å,^46^ which agree with experimental results of 2.368 Å,^47^ 2.54 Å,^48^ and 2.38 Å,^49^ respectively. Another side, a lot of satisfactory instances of ABCluster were presented lately.^22^ Therefore, the conclusions derived from the ABCluster search technique coupled with the mPW2PLYP functional should be reliable.

## 3. Results and discussion

### 3.1 Structures and evolutions of GdGe_*n*_^−^ compounds

**Fig. 1:**
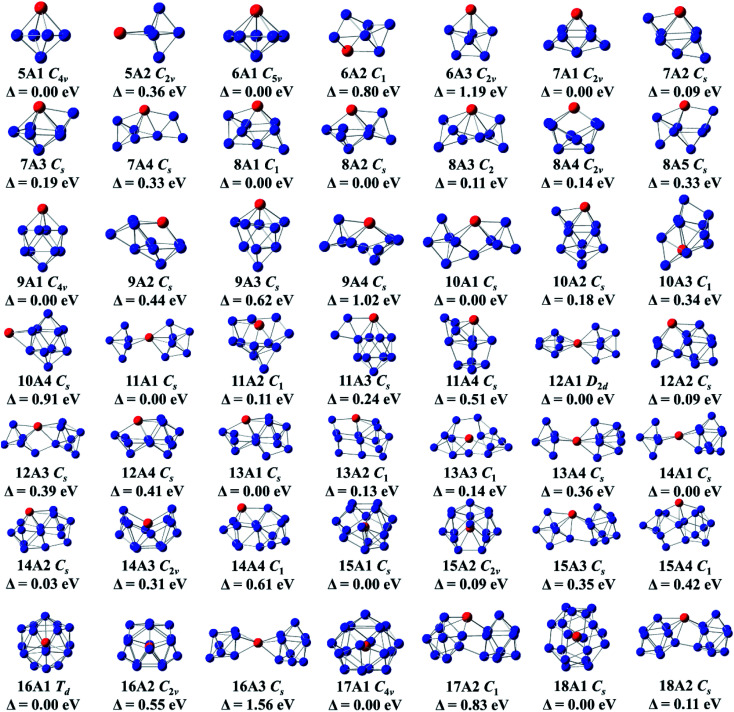
Lowest energy structure and isomers of GdGe_*n*_^−^ (*n* = 5–18) anionic nanoclusters, point group and relative energy (in eV). The blue and red circles represent germanium and gadolinium atoms, respectively.

All selected configurations, including most stable and low-lying configurations of doping Ge anionic clusters with Gd atom are displayed in Fig. 1. The compounds are designated as *n*A*m*, with *n* representing the number of Ge atoms, A representing anion, and *m* representing the number of compounds, based on their energies ranging from low to high. For GdGe_5_^−^ compound, two isomers are reported. Its global minimum structure is predicted to be *C*_4v_-symmetry *tetragonal bipyramid* (5A1) in ^8^A_2_ ground state. The *C*_2v_-symmetry *edge-capped trigonal bipyramid* (5A2) of ^8^A_2_ electronic state is above 0.36 eV than the 5A1 in energy. For GdGe_6_^−^ compound, there are three isomers which are presented here. The most stable structure is evaluated to be *C*_5v_-symmetry *pentagonal bipyramid* (6A1) in ^8^A_1_ ground state. Both compounds of *C*_2v_-symmetry *pentagonal bipyramid* (6A3) in ^8^B_2_ electronic state and *C*_1_-symmetry 6A2 are less stable in energy than that of 6A1 by 1.19 and 0.80 eV, respectively. For GdGe_7_^−^ compound, four structures are reported. The *C*_2v_-symmetry 7A1 in ^8^A_2_ ground state can be viewed by attaching two Ge atoms to the 5A1 structure. The *C*_s_-symmetry *bicapped octahedron* (7A-2) can be viewed as a Gd atom substituting for a Ge atom in the most stable structure of Ge_8_ compound.^13^ The *C*_s_-symmetry 7A3 can be regarded by attaching a Ge atom to the 6A1 geometries. The *C*_s_-symmetry 7A4 can be considered as linked structure in which Gd atom connects a Ge_3_*triangle* and a Ge_4_*tetrahedron*. In ^8^A′′ electronic state, they are 0.09, 0.19 and 0.33 eV higher in energy than that of 7A1, respectively. For GdGe_8_^−^ compound, five isomers are presented. The *C*_1_-symmetry 8A1 is predicted to be the global minimum structure. It can be viewed by attaching a Ge atom to a face of 7A1 geometry. The 8A2, 8A4 and 8A5 can be viewed as adding dual Ge atoms to the 6A1 geometry. They are *C*_s_-symmetry in ^8^A′′ electronic state, *C*_2v_-symmetry in ^8^A_2_ electronic state, and *C*_s_-symmetry in ^8^A′′ electronic state. The 8A3 geometry, similar to the most stable structure of GdSi_8_^−^ compound,^50^ is *C*_2_-symmetry with ^8^A electronic state. It belongs to linked structure in which Gd atom links two germanium *tetrahedral*. The linked structures were firstly proposed by Kumar and co-workers.^51^ Energetically, the 8A2, 8A3, 8A4 and 8A5 isomers are 0.08, 0.11, 0.14 and 0.33 eV higher than that of 8A-1, respectively. For GdGe_9_^−^ compound, four configurations are presented. The global minimum structure is calculated to be a *bicapped antitetragonal prism* (9A1) with *C*_4v_-symmetry and ^8^A_2_ ground state analogous to that of GdSi_9_^−^ compound.^50^ The 9A2 can be viewed by attaching a Ge_3_ to the ground state structure of GdGe_6_^−^ compound. The 9A2, 9A3, and 9A4 isomers have *C*_s_-symmetry with ^8^A′′ electronic state. They are 0.44, 0.62, and 1.02 eV higher in energy than that of 9A1, respectively.

For GdGe_10_^−^ compound, four structures are presented. The global minimum structure is forecasted to be 10A1 linked structure with *C*_s_-symmetry in ^8^A′′ ground state in which Gd atom connects a Ge_4_*tetrahedron* and a Ge_6_*capped trigonal bipyramid*. The *C*_s_-symmetry 10A2 of ^8^A′′ electronic state can be noted as capping the lowest energy isomer (9A1) of GdGe_9_^−^ by a Ge atom close to the metal atom. The *C*_1_-symmetry 10A3 can be viewed as substituting a Gd atom for a Ge atom in the ground state structure of Ge_12_.^13,45^ The *C*_s_-symmetry 10A4 of ^8^A′′ electronic state, Gd-*capped bicapped antitetragonal prism* of Ge_10_, is comparable to the that of LuGe_10_ compound.^17^ They are higher in energy than that of 10A1 by 0.18, 0.34, and 0.91 eV, respectively. For GdGe_11_^−^ compound, four geometries are reported. The ground state 11A1 is linked configuration where Gd atom links two sub-groups of Ge_5_ and a Ge_6_. 11A2 geometry can be considered as adding four Ge atoms to the face of 7A2 structure. The *C*_s_-symmetry 11A3 of ^8^A′′ state can be considered as adding Ge_2_ to the edge of the ground state *bicapped antitetragonal prism* of GdGe_9_^−^ compound. These compounds and *C*_s_-symmetry 11A4 of ^8^A′′ electronic state are less stable as compared with 11A1 by 0.11, 0.24, and 0.51 eV, respectively. For GdGe_12_^−^ complex, four geometries are noted. They are linked structures in which Gd links two orthogonal Ge_6_*distorted tetragonal bipyramid*, links a Ge_3_*isosceles triangle* and a Ge_9_*tricapped trigonal prism* (TTP). It also links a Ge_5_*trigonal bipyramid* and a Ge_7_*pentagonal bipyramid*, and links a Ge_4_*quadrilateral* and a Ge_8_*antitetragonal prism*, respectively. Energetically, *D*_2d_-symmetry 12A1 of ^8^A_2_ ground state is more stable than those of *C*_s_-symmetry in ^8^A′′ state about 0.09, 0.39, and 0.41 eV. respectively. For GdGe_13_^−^ compound, four geometries are presented. The *C*_1_-symmetry 13A1, *C*_s_-symmetry 13A11, 13A2 and 13A4 in ^8^A′′ state belong to linked shapes where Gd atom connects a Ge_4_*tetrahedron* and a Ge_9_ TTP, a Ge_4_*rhombus* and a Ge_9_ TTP, and a Ge_5_*trigonal bipyramid* with a Ge_8_ subcluster, respectively. The energy difference compared with the most stable structure of 13A1 is 0.13, 0.14, and 0.36 eV, respectively. For GdGe_14_^−^ compound, four configurations are described. Its most stable structure is calculated to be Gd-linked motif (14A1) with *C*_s_-symmetry and ^8^A′′ state where Gd atom attaches a Ge_5_ and a Ge_9_ motif. The *C*_s_-symmetry 14A2 in ^8^A′′ state belongs to a linked structure where Gd atom joins a Ge_5_*tetragonal pyramid* and a Ge_9_ TTP. The *C*_2v_-symmetry 14A3 in ^8^A_2_ state is a semi-cage configuration. The 14A4 can be viewed by replacing a Ge atom in Ge_15_ ground state compound^14^ by Gd atom. The 14A1 is more stable than those of 14A2, 14A3 and 14A4 by 0.04, 0.31, and 0.61 eV, respectively.

For GdGe_15_^−^ compound, four motifs are presented. The first two isomers are Gd-encapsulated frameworks. The 15A1 cage can be considered as being derived from fullerene of Ge_16_ through removing a Ge atom, called as f-cage framework. Also, the 15A2 cage can be regarded as being derived from Frank–Kasper (FK) cage of Ge_16_ by eliminating a Ge atom, called as FK-cage framework. The f- and FK-cage frameworks possess *C*_s_-symmetry with ^8^A′′ ground state and *C*_2v_-symmetry in ^8^A_2_ state. The f-cage framework is more stable than that of FK-cage by 0.09 eV in energy. The latter two isomers are Gd-linked structures in which Gd atom links a Ge_6_ subunit and a Ge_9_ TTP motif. They are 0.35 and 0.42 eV higher than that of f-cage framework in energy, respectively. For GdGe_16_^−^ compound, three isomers are reported. The *T*_d_-symmetry Gd-encapsulated FK-cage in ^8^A_1_ ground state is the most stable configuration. *C*_2v_-symmetry f-cage endohedral framework (16A2) of ^8^A_2_ electronic state is less stable in energy than 16A1 by 0.55 eV. The 16A3 with *C*_s_-symmetry and ^8^A′′ electronic state is 1.56 eV higher in energy than that of FK-cage framework. For GdGe_17_^−^ compound, two isomers are presented. One of them is Gd-encapsulated five-capped FPTQ (four pentagonal faces and two quadrangles) cage framework (17A1) with *C*_4v_-symmetry and ^8^A_2_ ground state. It is more stable in energy than the *C*_1_-symmetry 17A2 linked geometry by 0.83 eV. For GeGe_18_^−^ compound, two structures are presented. The most stable geometry is Gd-encapsulated endohedral configuration (18A1) with *C*_s_-symmetry in ^8^A′′ ground state, of which energy is lower than that of 18A2 linked structure with *C*_s_-symmetry and ^8^A′′ electronic state by 0.11 eV.

Before the discussion of most stable structure, we concentrate on the structural transformation of GdGe_*n*_^−^ (*n* = 5–18) compounds at present. In the light of their structural characteristic of the determined global minimum configuration, the structural evolution favors Gd-linked configuration where metal atom connects two Ge subclusters starting from *n* = 10, and Gd-encapsulated germanium cage-like configuration is favored when *n* reaches to 15. Compared with LuGe_*n*_^−^ (*n* = 5–17) clusters,^11^ except for the different electronic states (the ground states of LuGe_*n*_^−^ (*n* = 5–17) clusters are singlet), the most stable geometries of GdGe_*n*_^−^ with *n* = 8, 10, and 15 are different from those of LuGe_*n*_^−^ clusters.

### 3.2 Magnetic moment and charge transfer

To learn more about the interaction between Gd atom and germanium nanoclusters, NPA of the GdGe_*n*_^−^ (*n* = 5–18) global minimum structure is carried out. The results including NPA configurations and NPA charges on Gd atom, the 4f, 5d, 6s, 6p and total magnetic moments of Gd, and total magnetic moments of compounds are shown in Table S2 in ESI.† It can be seen from Table S2† that (i) in GdGe_*n*_^−^ (*n* = 5–18) compounds have the valence configuration of Gd as 6s^0.36–0.81^4f^6.99–7.00^5d^1.71–5.30^6p^0.25–2.05^ which discloses that the 4f electrons of Gd ([Xe]6s^2^4f^7^5d^1^) hardly participate in bonding. This phenomenon differs from Tb atom in TbSi_*n*_^−^ compounds where 4f electrons of Tb prefer to take part in bonding *via* the 4f electrons jump into 5d orbitals.^52^ (ii) Because there are no 4f electrons involved in bonding, the oxidation number of the Gd in GdGe_*n*_^−^ compounds is three. (iii) For *n* = 5–9, the charge of Gd in GeGe_*n*_^−^ is within +0.15 to +0.37 a.u. It indicates that Gd is an electron donor, so the ionic bonds between Gd and germanium skeleton may be dominant. For cage-like configurations (*n* = 15–18), the charge of Gd is from −3.17 to −4.87 a.u., demonstrating that Gd is an electron acceptor and the bond nature between Gd and the host of the germanium cluster may be principally metallic bonds. And for linked structures (*n* = 10–15), the charge of Gd is from +0.25 to −0.26 a.u., revealing the fact that the characteristics of bonding between Gd and germanium clusters may be mixed with ionic bonds and covalent bonds in essence. (iv) The total magnetic moments of GdGe_*n*_^−^ (*n* = 5–18) compounds are kept at the value of 7 *μ*_B_, and provided by the 4f electrons of Gd atom which are left nearly unperturbed.

### 3.3 Stability

Average atomization energy (AAE) and second energy difference (Δ^2^*E*) as two substantial parameters to evaluate thermodynamic and relative stability, have been performed on the most stable structures of GdGe_*n*_^−^ (*n* = 5–18) compounds *via* atomization and disproportional reaction as follow:

1 GdGe_*n*_^−^ → (*n* − 1)Ge + Ge^−^ + Gd

2 2GdGe_*n*_^−^ → GdGe_*n*+1_^−^ + GdGe_*n*−1_^−^

**Fig. 2:**
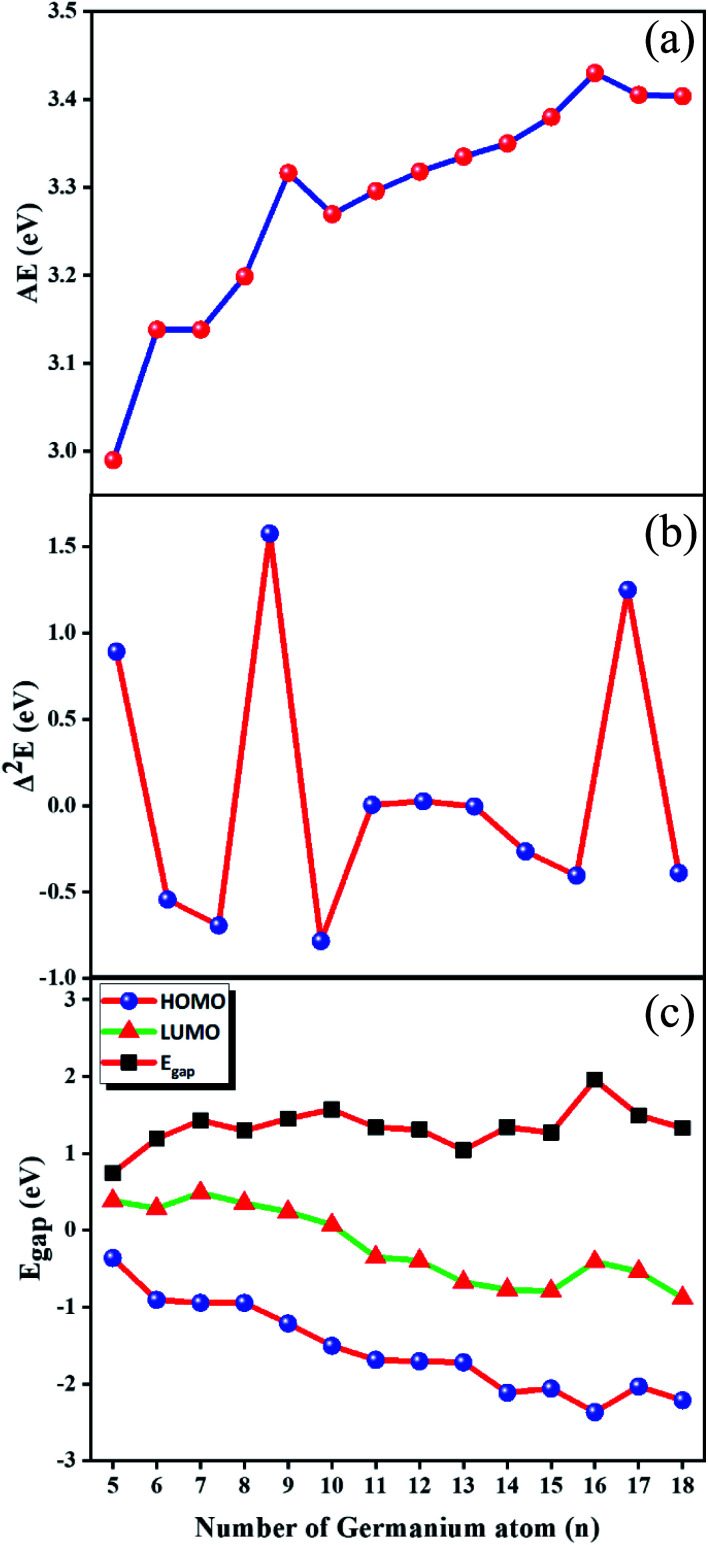
Size dependences of (a) average atomization energy (AAE); (b) second energy difference (Δ^2^*E*); and (c) HOMO–LUMO energy gap (*E*_gap_).

Incremental AAE is an effective approach to examine the local relative stability of different size compounds. The AAE of GdGe_*n*_^−^ (*n* = 5–18) compounds as a function of the size of the compound is shown in Fig. 2(a), from which it can be deduced that GdGe_9_^−^ and GdGe_16_^−^ compounds are more stable than proposed by flat rising background. In addition to AAE, Δ^2^*E* can not only mirror the local relative stability, but also gives a susceptible measure as shown in Fig. 2(b). The larger the Δ^2^*E*, the stronger the relative stability. The results of AAE are clearly reproduced in Fig. 2(b). It is noted that GdGe_9_^−^ compound has only good relative stability, not the best thermodynamic stability. However, GdGe_16_^−^ compound not only has good relative stability, but also has the best thermodynamic stability due to the fact that its AAE is the largest.

Compared to anionic LuGe_*n*_^−^ (*n* = 5–17) clusters,^11^ the AAE curves of LuGe_*n*_^−^ and GdGe_*n*_^−^ are in parallel as can be seen from Fig. S2 in ESI.† And the AAE values of LuGe_*n*_^−^ clusters are slightly larger than those of GdGe_*n*_^−^ by 0.04 eV on average, indicating the stability of anionic germanium clusters doped with 4f orbital fully-filled Lu atom is slightly better than that of doped with 4f orbital half-filled Gd atom.

### 3.4 HOMO–LUMO energy gaps

An important physical parameter closely involved in chemical stability is HOMO–LUMO energy gap (*E*_gap_). In the *E*_gap_ quantitative evaluation, Baerends *et al.*^53^ mentioned that the *E*_gap_ calculated *via* pure density functional theory (DFT) is closer to the real optical gap than that evaluated by hybrid DFT due to the fact that the energy of HOMO and LUMO predicted in Kohn–Sham molecular orbital approximations experience in general the alike quantity increase. However, HF approach moves the LUMO up a much higher energy levels than the HOMO up, which results in the *E*_gap_ of hybrid DFT becomes larger than that of pure DFT. Recently, An Wei^15^ calculated the *E*_gap_ of Ge_*n*_ (3 ≤ *n* ≤ 20) by using the PBE scheme, compared them with experiment data, and found that the theoretical *E*_gap_ match well with those in experiment. Therefore, the PBE scheme^39^ are employed to evaluate the *E*_gap_ of GdGe_*n*_^−^ (*n* = 5–18) compounds. The used basis sets are aug-cc-pVTZ^32^ and def2-TZVP^29,30^ for Ge and Gd atoms, respectively. They along with energies of HOMO and LUMO are shown in Fig. 2(c). We can see from it that the *E*_gap_ of GdGe_*n*_^−^ (*n* = 5–18) compounds range from 0.75 to 1.96 eV. GdGe_5_^−^ compound has the smallest *E*_gap_ (0.75 eV) because it has a relatively high HOMO energy. While GdGe_16_^−^ compound possesses the largest *E*_gap_ (1.96 eV) due to the fact that it has the lowest HOMO energy level. The larger the *E*_gap_, the better the chemical stability. It is proved that the GdGe_16_^−^ cluster assembly material has ideal thermodynamic stability, chemical stability and energy gap, so that it would become an excellent semiconductor material. Compared to the *E*_gap_ of LuGe_*n*_^−^ (*n* = 5–17) clusters, the *E*_gap_ of GdGe_*n*_^−^ for *n* = 5, 6, 11–13, and 15–17 differs little from that of LuGe_*n*_^−^ clusters, and for *n* = 7–10, and 14, the difference is between 0.16–0.69 eV as can be seen from Fig. S3 in ESI.†

### 3.5 PES of GdGe_*n*_^−^ compounds

**Fig. 3:**
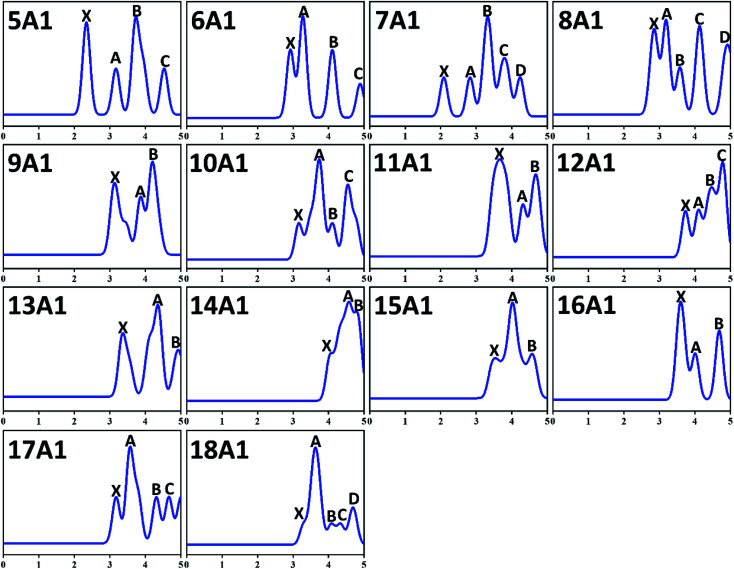
Simulated PES spectra of the ground-state structures of GdGe_*n*_^−^ (*n* = 5–18) nanoclusters.

Spectral information is of considerable importance because the PES is an exceedingly hypersensitive approach for examining both electronic structures and equilibrium configuration of anionic atom, molecules and compounds. In particular, there is no experimental approach for directly determining the ground state configuration of compounds by now. One can only indirectly determine the ground state structures *via* detailed comparison of theoretical and experimental results. And PES is one of the most effective strategies. Therefore, we simulated the PES of GdGe_*n*_^−^ (*n* = 5–18) compounds in order to provide strong motivation and theoretical information for future experimental investigations. In the PES simulation, to fit all peaks in the region of less than 5.00 eV, a Gaussian FWHM of 0.25 eV is utilized. The theoretical PES spectra are shown in Fig. 3. From it, we can see that for *n* = 5 and 6, four distinct peaks (X, A, B, C) are resided at 2.34, 3.18, 3.73, and 4.55 eV, and 2.92, 3.27, 4.11, and 4.89 eV, respectively. For *n* = 7 and 8, there are five peaks (X, A–D) resided at 2.08, 2.82, 3.33, 3.79, and 4.24 eV, and 2.84, 3.19, 3.57, 4.11 and 4.91 eV, respectively. Among them, the VDE of GdGe_7_^−^ (2.08 eV) is the smallest among these investigated compounds. For *n* = 9, three obvious peaks located at 3.10, 3.87, and 4.19 eV are observed. The simulated PES of GdGe_10_^−^ and GdGe_12_^−^ exhibit four peaks (X, A–C) centered at 3.16, 3.73, 4.11 and 4.55 eV, and 3.73, 4.10, 4.46 and 4.78 eV, respectively. Three obvious peaks (X, A, B) for GdGe_11_^−^, GdGe_13_^−^ and GdGe_14_^−^ are simulated at 3.63, 4.32 and 4.66 eV, 3.34, 4.34 and 4.91 eV, and 4.03, 4.56 and 4.77 eV, respectively. The VDE of GdGe_14_^−^ (4.03 eV) is the largest among these investigated compounds. And it is a weaker shoulder peak. There are also three obvious peaks (X, A, B) for *n* = 15 and 16. They are resided at 3.48, 4.01, 4.57 eV, and 3.59, 4.01, 4.68 eV, respectively. For *n* = 17, four obvious peaks (X, A–C) located at 3.18, 3.57, 4.31 and 4.66 eV are observed. For *n* = 18, the first peak (X) resided at 3.29 eV is a weaker shoulder. Its third and fourth peaks (B and C) are also relatively weaker peaks resided at 4.09 and 4.34 eV, respectively. The second and fifth peaks (A and D) centered at 3.62 and 4.69 eV are resolved easily. There are no experimental counterparts for comparison. We hope that our theoretical simulations will provide great incentive for further experimental research on these crucial Gd-doped germanium nanoalloys.

### 3.6 Infrared and Raman spectra

**Fig. 4:**
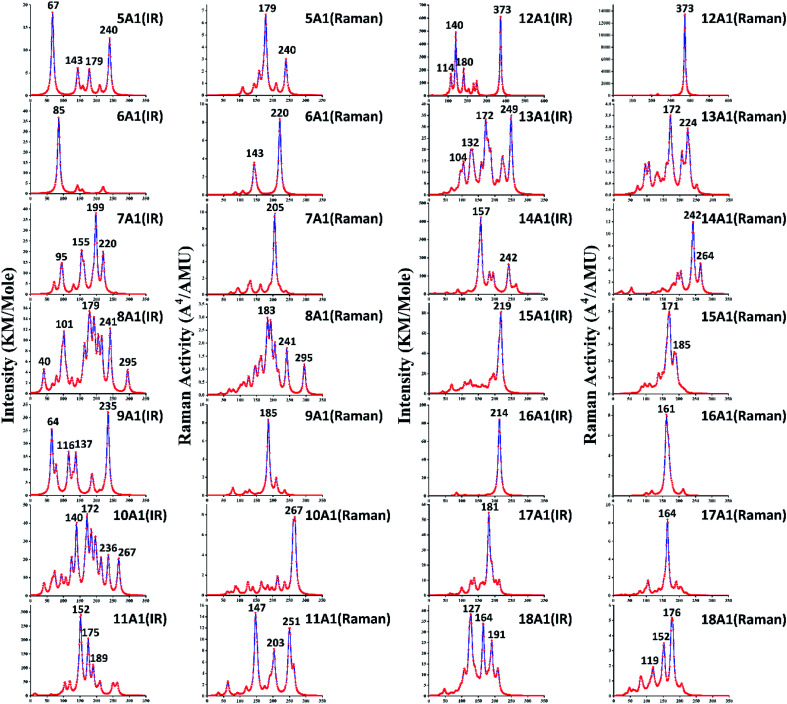
Infrared and Raman spectra of GdGe_*n*_^−^ (*n* = 5–18) nanoclusters.

In addition to PES, infrared and Raman spectra are also one of the effective schemes to indirectly determine the ground state structures. The infrared and Raman spectra of GdGe_*n*_^−^ (*n* = 5–18) compounds have been computed using the PBE0 method to better understand their vibrational features. The basis set used are aug-cc-pVTZ and def2-TZVP for Ge and Gd atoms, respectively. They are shown in Fig. 4 where no imaginary frequency was observed, which demonstrates that the structure is stable. In the infrared and Raman spectra of the GdGe_5_^−^ compound, there are four and two prominent peaks observed, respectively. An angle-bending is doubly degenerated vibration mode at 67 cm^−1^, and it leads to the highest intense infrared frequency. The second lowest vibration mode at 143 cm^−1^ with infrared active is breathing mode of GdGe_5_*bipyramid*. The vibration modes at 179 cm^−1^ and 240 cm^−1^ with Raman and infrared active are breathing mode of LuGe_5_*tetragonal bipyramid* and stretching mode of Ge_5_*tetragonal pyramid* respectively. For GdGe_6_^−^ compound, only one resolved infrared peak at 85 cm^−1^ is doubly degenerated angle-bending vibration mode. Two vibration modes at 143 and 220 cm^−1^ in Raman spectra are stretching mode of GdGe_5_*pentagonal pyramid* and breathing mode of Ge_6_*pentagonal pyramid* respectively. In infrared and Raman spectra of GdGe_7_^−^ compound, four and one prominent peaks are seen, respectively. The vibration modes at 95 cm^−1^ and 155 cm^−1^ belong to the bending mode of GdGe_7_, that at 199 cm^−1^ and 220 cm^−1^ belong to the stretching mode of GdGe_7_, and that at 205 cm^−1^ of Raman spectra is the breathing mode of GdGe_7_. In infrared and Raman spectra of GdGe_8_^−^ compound, five and three prominent peaks are seen, respectively. The strongest peak in infrared spectra is at 179 cm^−1^, which is resulted from the stretching mode of the Ge_4_*tetrahedron*, and that in Raman spectra is at 183 cm^−1^, which is resulted from the breathing mode of the GdGe_8_. In infrared and Raman spectra of GdGe_9_^−^ compound, there are four and one prominent peaks, and the strongest peaks locate at 235 cm^−1^ and 185 cm^−1^ with breathing and stretching mode of GdGe_9_, respectively. Three lowest vibration modes of 64, 116 and 137 cm^−1^ are doubly degenerated bending vibration mode.

In infrared and Raman spectra of GdGe_10_^−^ compound, four and one prominent peaks are respectively reported. The vibration mode at 267 cm^−1^ with Raman and infrared active is breathing mode of Ge_4_*tetrahedron*. The most prominent peak in infrared spectra at 172 cm^−1^ results from the breathing mode of the Gd-linked Ge_4_*tetrahedron* and Ge_6_*capped trigonal bipyramid* together. The second most prominent peak in infrared spectra at 140 cm^−1^ is the breathing mode of Gd-linked Ge_6_. The infrared vibration mode at 236 cm^−1^ is the stretching mode of Gd_6_*capped trigonal bipyramid*. In infrared and Raman spectra of GdGe_11_^−^ compound, three prominent peaks are reported. The two most intense infrared peaks at 152 and 175 cm^−1^ are resulted from the breathing mode of Ge_6_ and Gd-linked Ge_5_ together. The vibration mode at 189 cm^−1^ with infrared active is breathing mode of Gd-linked Ge_5_. The strongest peak in Raman spectral located at 147 cm^−1^ is bending mode of Gd-linked Ge_6_. The vibration mode at 251 cm^−1^ in Raman spectra results from the breathing mode of Ge_6_, and that at 203 cm^−1^ results from doubly degenerated stretching vibration mode. In infrared and Raman spectra of GdGe_12_^−^ compound, the most prominent peak at 373 cm^−1^ results from the doubly degenerated breathing mode of Gd-linked Ge_6_. In addition, there are three dominant peaks in infrared spectra at 114, 140 and 180 cm^−1^ related to bending mode of GdGe_12_. In infrared and Raman spectra of GdGe_13_^−^ compound, four and three prominent peaks are seen. The most intense peak in infrared spectra at 249 cm^−1^ results from stretching mode. The second most intense peak in infrared and the most intense peak in Raman located at 172 cm^−1^ results from bending mode of GdGe_13_. The vibration modes at 104 cm^−1^ and 132 cm^−1^ with infrared active are stretching and bending mode respectively. And that at 224 cm^−1^ in Raman spectra results from bending mode. For GdGe_14_^−^ compound, two dominant peaks are reported. The most prominent peak in infrared spectra at 157 cm^−1^ results from the breathing mode of Gd-linked Ge_5_*trigonal bipyramid*. In Raman and infrared spectra, the vibration mode at 242 cm^−1^ results from the breathing mode of Gd-linked Ge_9_ TTP, and that in Raman spectra at 264 cm^−1^ is the breathing mode of Ge_5_*trigonal bipyramid*.

For GdGe_15_^−^ compound, only one prominent peak at 219 cm^−1^ in infrared spectra arises from the doubly degenerated bending mode. There are two major peaks in the Raman spectra at 171 cm^−1^ and 185 cm^−1^ in the bending mode of Gd-doped Ge_15_ motif and the breathing mode of peripheral Ge cage configuration (Gd atom remains static), respectively. In infrared and Raman spectra of GdGe_16_^−^ compound, only one main peak resides at 214 cm^−1^ with the threefold degenerate bending mode and 161 cm^−1^ for breathing mode of peripheral Ge cage (Gd atom motionless). For GdGe_17_^−^ compound, there is also single peak in infrared and Raman spectra, which resides respectively at 181 cm^−1^ with the doubly degenerate bending mode and 164 cm^−1^ in breathing mode. In infrared and Raman spectra of GdGe_18_^−^ nanocluster, there are three prominent peaks respectively. The most intense peak in infrared spectra resides at 127 cm^−1^ consisted of approximately triple times of degenerate bending mode. In infrared spectra the vibration modes at 164 cm^−1^ and 191 cm^−1^ are stretching mode. In Raman spectra the largest peak resides at 176 cm^−1^ with the approximately doubly degenerated breathing mode, and those at 119 cm^−1^ and 152 cm^−1^ are bending mode.

As we could know from the description above, infrared and Raman activity manifests different spectra for these compounds and reflects the influence of geometrical changing. According to infrared analysis, breathing mode of the Gd-linked Ge subclusters for Gd-linked configurations excluded GdGe_13_^−^ compound give rise to the most intense peak, and it is degenerated bending mode for Gd-encapsulated frameworks. In Raman spectra, the strongest peak is largely breathing or bending mode of Ge subclusters or Gd-linked Ge subclusters for Gd-linked geometries, and it is breathing mode of peripheral Ge cage (Gd atom hardly moves) for Gd-encapsulated structures. They occur in the infrared range of these compounds in comparison with the 400–10 cm^−1^ far-infrared region. Therefore, the most stable compounds with component might be useful for far-infrared sensing devices.

### 3.7 Iso-chemical shielding surface of GdGe_16_^−^ compound

**Fig. 5:**
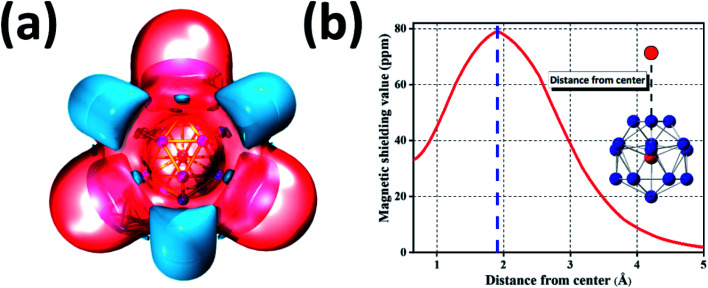
ICSS of GdGe_*n*_^−^ nanocluster. (a) Calculated ICSS isosurface with isovalue of 0.05 ppm (red region) and −0.05 ppm (blue region); (b) ICSS curve line of magnetic shielding value with distance from center.

Because of great potential application of GdGe_16_^−^ nanocluster in optoelectronic devices, we further evaluated its stability *via* the method of iso-chemical shielding surface (ICSS), which is the negative value of nuclear independent chemical shielding (NICS), and was carried out by gauge-independent atomic orbital (GIAO) way.^54,55^ In Fig. 5(a), it is displayed that the whole real space displays the red region that means the chemical shielding opposed the external magnetic field with the isovalue of 0.05 ppm, and the blue region represents the chemical deshielding area with the isovalue of −0.05 ppm. Both of them have symmetry because GdGe_16_^−^ nanocluster has a high symmetry of *T*_d_. Clearly, inner cage area has a larger chemical shielding effect and outer has *vice versa*. In the Fig. 5(b), the curve map shows one direction shielding value which relates to the distance. Generally, the shielding value in the distance of 1 angstrom is a standard parameter to evaluate the aromaticity of the system, *i.e.*, ICSS(1) = 46 ppm. Besides that, the maximum shielding value is 78 ppm in a distance of 1.91 Å. In short, the stability of GdGe_16_^−^ nanocluster has been revealed by the ICSS methods. Moreover, the excellent stability of such cluster has been further proved.

**Fig. 6:**
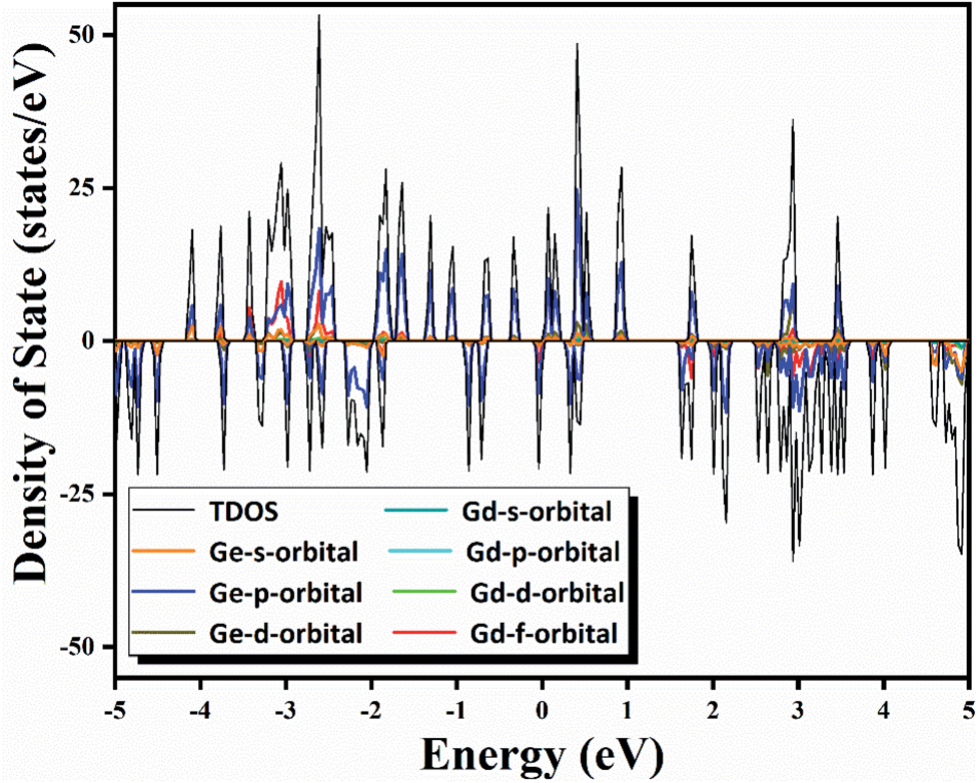
Calculated total and partial density of states of GdGe_16_^−^ nanocluster.

To further understand the outstanding chemical and thermal stabilities of GdGe_16_^−^ nanocluster, the total and partial density of states analysis are shown in Fig. 6. In the near Fermi level of which the most contribution belongs to the 4p orbital of Ge atom which was mixed with the major of 5d and 6s orbital of Gd atom to form the hybrid bonds which stabilizes the whole structure. In the whole range, the spin up curve and spin down curve are asymmetric that indicates the system has magnetism and spin polarization effect. Combined with NPA analysis, we have known that Gd 4f electrons in half-filled state do not participate in the bonds, and hence provide magnetism. The total valence of 75 electrons of GdGe_16_^−^ system can be distributed to the orbital sequence of 1S^2^1P^6^(4f^7^)1D^10^1F^14^2S^2^2P^2^1G^18^2P^4^2D^10^, which complies with not only Hund's rule, but also spherical jellium model. Hence, it proves that GdGe_16_^−^ nanocluster is a superatom.

### 3.8 UV-Vis spectra of GdGe_16_^−^ molecule

**Fig. 7:**
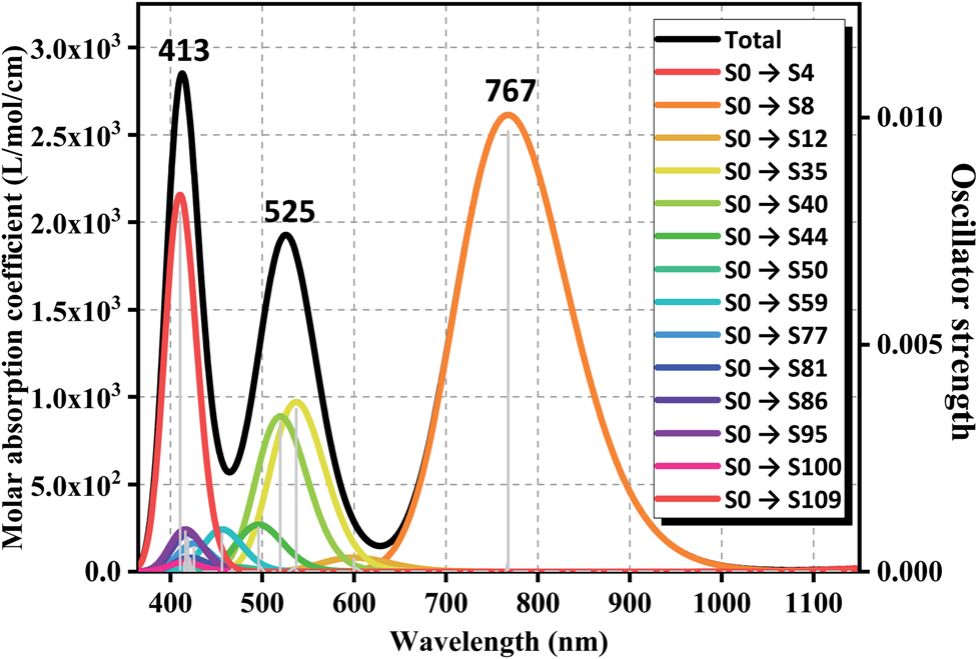
Simulated UV-Vis spectrum of GdGe_16_^−^ superatom. Solid and dotted lines stand for the absorption curve and oscillator strength, respectively.

Owing to the high stability and proper semiconductor characteristics of anion cluster of GdGe_16_^−^, the ultraviolet-visible (UV-Vis) spectra have been simulated by time-dependent density functional theory (TD-DFT) calculation by the PBE scheme with aug-cc-pVDZ and ECP28MWB basis set for Ge and Gd atoms respectively. To ensure the accuracy of calculation, enough bands were required to be considered, so the 120 excited states were performed to satisfy the described system. Full results are assembled in Fig. 7 with the Gaussian broadening value of 0.30 eV. Overall, the UV-Vis absorption spectrum of GdGe1_6_^−^ anion produces three absorption bands, two of them fall in the visible region and one in the near-infrared region. Compared to UV-Vis spectra of LuGe_16_^−^,^11^ the UV-Vis spectra of GdGe_16_^−^ have obvious red shift. The first absorption band is from 350 nm to 465 nm. The strongest peak is at 413 nm. The second absorption band, with the strongest peak at 525 nm, is from 465 nm to 628 nm. The third absorption band having range of 628 nm to 1050 nm has the most intense peak at 767 nm. For summit of 413 nm, it is made of S_0_ → S_109_, S_0_ → S_95_, S_0_ → S_86_ with the contribution of 74.9%, 8.4%, 7.1%, respectively. For the peak of 525 nm, it is composed of S_0_ → S_35_, S_0_ → S_40_, S_0_ → S_44_ with the contribution of 46.7%, 45.1%, 7.6%, respectively. The last peak of 767 nm is attributed to the transition of 99% of S_0_ → S_9_. As we know, the solar energy distribution is 43% visible light with the most intensity, and 52% near-infrared with the energy intensity gradually decreased by increased wavelength so that the GdGe_16_^−^ nanocluster can utilize most of the solar energy because its broad absorption ranges match well with solar energy distribution. That is to say, such material is potential candidates of solar energy converter or ultra-highly sensitive near-infrared photodetector.

## 4. Conclusion

All in all, the structural evolution of Gd-doped germanium anionic compounds, GdGe_*n*_^−^ (*n* = 5–18) has been explored *via* quantum chemistry calculations using mPW2PLYP method and unprejudiced structural searching technique ABCluster. The results have clearly shown that with the increase of cluster size *n*, the structure evolution pattern moves from the Gd-linked configuration (*n* = 10–14) in which the Gd acts as a linked (the Gd atom links two germanium sub-clusters) to the Gd-encapsulated form (*n* = 15–18) in which the Gd atom is resided in the center of the germanium cage. The properties including magnetic moment, charge transfer, relative stability, HOMO–LUMO gap, PES, infrared and Raman spectra have been reported. The information of these spectra could give extra approaches to experimentally determine the electronic structures and equilibrium configuration of these compounds. The largest spin magnetic moment of 7 *μ*_B_ for these species is attained *via* half-filled 4f states. The GdGe_16_^−^ nanocluster is a superatom due to the fact that its total valence of 75 electrons can be distributed to the orbital sequence of 1S^2^1P^6^(4f^7^)1D^10^1F^14^2S^2^2P^2^1G^18^2P^4^2D^10^, which complies with not only Hund's rule, but also with the spherical jellium model. Particularly, its UV-Vis spectra match well with solar energy distribution. Such materials can act as nano multifunctional building units which are potentially used in solar energy converter or ultra-highly sensitive near-infrared photodetector.

## Conflicts of interest

The authors declare no conflict of interest.

## Supplementary Material

RA-012-D2RA04037A-s001
