# Supplementary material for: Structural evolution, photoelectron spectra and vibrational properties of anionic GdGen− (n = 5–18) nanoalloy clusters: a DFT insight
Source: RSC Adv. 2022 Aug 10;12(34):22020–30. doi: 10.1039/d2ra04037a (PMC9364359; doi:10.1039/d2ra04037a)
Supplement: RA-012-D2RA04037A-s001 [file RA-012-D2RA04037A-s001.pdf]

Electronic Supplementary Material (ESI) for RSC Advances.
This journal is © The Royal Society of Chemistry 2022

# Supplementary Information

## Structural Evolution, Photoelectron Spectra and Vibrational Properties of Anionic $GdGe_n^-$ ($n$=5-18) Nanoalloy Clusters: A DFT Insight

Zhaofeng Yang[a], Aziz U Rehman[a], Zhenzhu Cao[a], Jucai Yang[a,b*]

[a] School of Chemical Engineering, Inner Mongolia University of Technology, and Inner Mongolia Key Laboratory of Theoretical and Computational Chemistry Simulation, Hohhot 010051, Peoples Republic of China

[b] School of Energy and Power Engineering, Inner Mongolia University of Technology, Hohhot 010051, Peoples Republic of China

*Jucai Yang E-mail: yangjc@imut.edu.cn

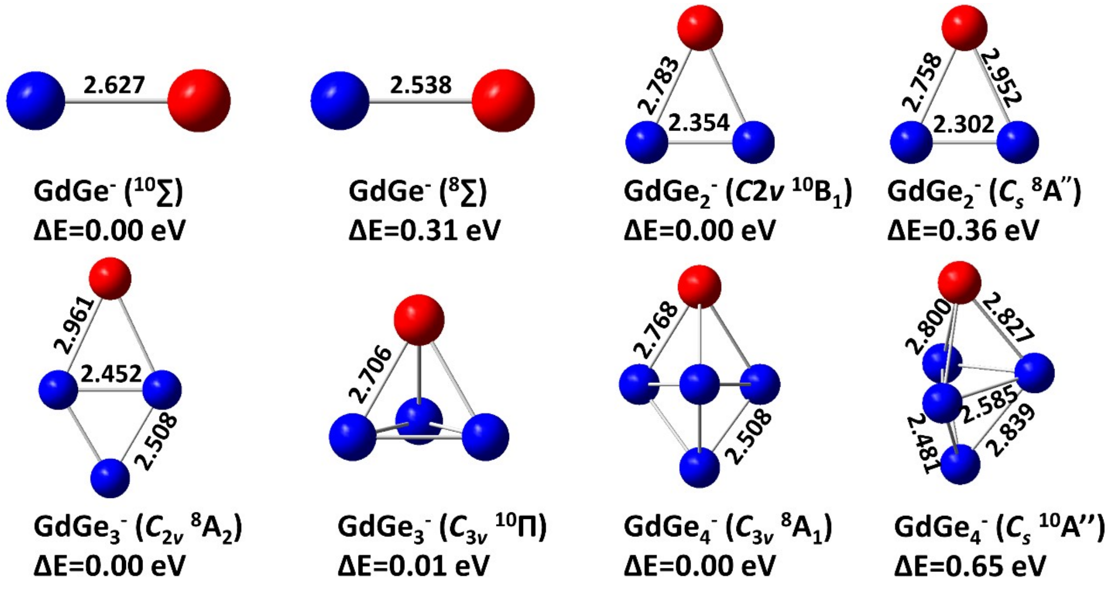


**Figure S1**. Lowest energy structure and isomers of $GdGe_n^-$ ($n$=1-4) anionic clusters, point group, electronic state and relative energy (in eV) calculated at mPW2PLYP/aug-cc-pVTZ,def2-TZVP//mPW2PLYP/cc-pVTZ-PP,def2-TZPV level. The blue and red circles stand for germanium and gadolinium atoms, respectively.

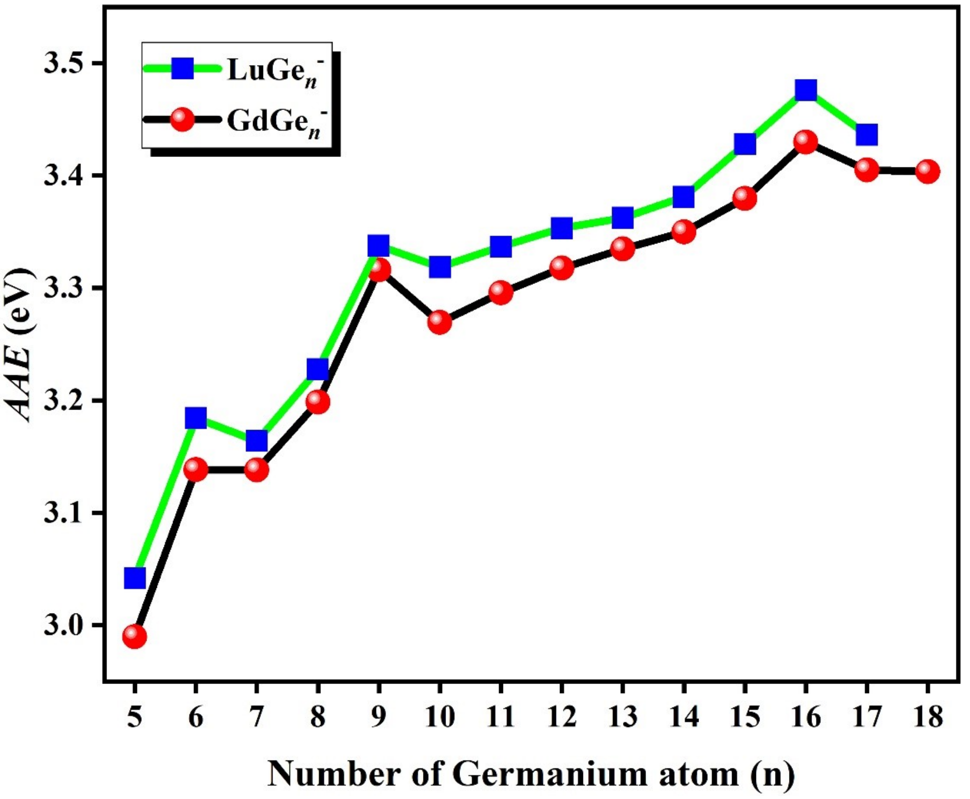


**Figure S2**. Size dependences of average atomization energy (AAE) for $GeGe_n^-$ and $LuGe_n^-$ ($n$=5-17) clusters. The values of $LuGe_n^-$ clusters are taken from Ref. 11.

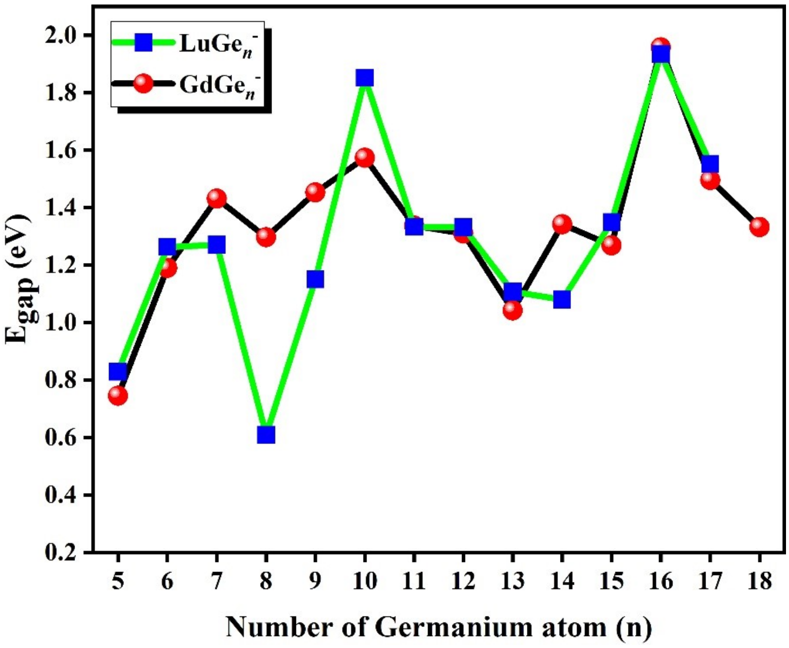


**Figure S3**. Size dependences of HOMO-LUMO energy gap (Egap) for $GeGe_n^-$ and $LuGe_n^-$ ($n$=5-17) clusters. The values of $LuGe_n^-$ clusters are taken from Ref. 11.

.

**Table S1.** Total energies (a.u.) of $GdGe_n^-$ ($n$=5-18) clusters in octuplet and decuplet calculated by using mPW2PLYP/(aug-cc-pVTZ,def2-TZVP)//mPW2PLYP/(cc-pVTZ-PP,def2-TZPV) scheme.

| | State | | | State | |
|---|---|---|---|---|---|
| Isomer | Octuplet | Decuplet | Isomer | Octuplet | Decuplet |
| **5A1** | -11148.59661 | -11148.54910 | **11A4** | -23609.02991 | -23609.00713 |
| **5A2** | -11148.58321 | -11148.57607 | **12A1** | -25685.78992 | -25685.74058 |
| **6A1** | -13225.35432 | -13225.31848 | **12A2** | -25685.78645 | -25685.72747 |
| **6A2** | -13225.32490 | -13225.29891 | **12A3** | -25685.77560 | -25685.73268 |
| **6A3** | -13225.31069 | -13225.31720 | **12A4** | -25685.77503 | -25685.75326 |
| **7A1** | -15302.07925 | -15302.02559 | **13A1** | -27762.53028 | -27762.49715 |
| **7A2** | -15302.07592 | -15302.03822 | **13A2** | -27762.52556 | -27762.48418 |
| **7A3** | -15302.07234 | -15302.07213 | **13A3** | -27762.52522 | -27762.48141 |
| **7A4** | -15302.06729 | -15302.01301 | **13A4** | -27762.51718 | -27762.45367 |
| **8A1** | -17378.82419 | -17378.77772 | **14A1** | -29839.27081 | -29839.21985 |
| **8A2** | -17378.82139 | -17378.79228 | **14A2** | -29839.26963 | -29839.21702 |
| **8A3** | -17378.82024 | -17378.77767 | **14A3** | -29839.25949 | -29839.24956 |
| **8A4** | -17378.81909 | -17378.80639 | **14A4** | -29839.24836 | -29839.22125 |
| **8A5** | -17378.81196 | -17378.78227 | **15A1** | -31916.02109 | -31915.96955 |
| **9A1** | -19455.59466 | -19455.53751 | **15A2** | -31916.01794 | -31915.96750 |
| **9A2** | -19455.57832 | -19455.51909 | **15A3** | -31916.00826 | -31915.95910 |
| **9A3** | -19455.57191 | -19455.51415 | **15A4** | -31916.00582 | -31915.96282 |
| **9A4** | -19455.55727 | -19455.51576 | **16A1** | -33992.78627 | -33992.71106 |
| **10A1** | -21532.30723 | -21532.25069 | **16A2** | -33992.76593 | -33992.72447 |
| **10A2** | -21532.30063 | -21532.27283 | **16A3** | -33992.72899 | -33992.68894 |
| **10A3** | -21532.29482 | -21532.26481 | **17A1** | -36069.50557 | -36069.44537 |
| **10A4** | -21532.27367 | -21532.26768 | **17A2** | -36069.47500 | -36069.43183 |
| **11A1** | -23609.04865 | -23608.99830 | **18A1** | -38146.23920 | -38146.18444 |
| **11A2** | -23609.04476 | -23609.00664 | **18A2** | -38146.23532 | -38146.21333 |
| **11A3** | -23609.03997 | -23608.98703 | | | |

**Table S2**. Natural population analysis (NPA) charge (in a.u.), valence configuration, magnetic moment (μB) of 6*s*, 4*f*, 5*d*, 6*p*, and total of Gd atom, and total magnetic moment of the global minima $GdGe_n^-$ ($n$=5-18) compounds calculated at the mPW2PLYP level.

| Compound | Charge (a.u.) | Electron Configuration | Magnetic Moment of Gd Atom 6*s* | 4*f* | 5*d* | 6*p* | Total | Molecule (μB) |
|---|---|---|---|---|---|---|---|---|
| $GdGe_5^-$ | 0.16 | [core]$6s^{0.81}4f^{7.00}5d^{1.71}6p^{0.32}$ | 0.01 | 6.98 | 0.24 | 0.01 | 7.24 | 7 |
| $GdGe_6^-$ | 0.25 | [core]$6s^{0.70}4f^{7.00}5d^{1.72}6p^{0.32}$ | 0.00 | 6.98 | 0.24 | 0.00 | 7.22 | 7 |
| $GdGe_7^-$ | 0.15 | [core]$6s^{0.57}4f^{7.00}5d^{2.03}6p^{0.25}$ | 0.03 | 6.98 | 0.17 | 0.00 | 7.18 | 7 |
| $GdGe_8^-$ | 0.15 | [core]$6s^{0.50}4f^{7.00}5d^{2.03}6p^{0.33}$ | 0.01 | 6.98 | 0.25 | 0.01 | 7.25 | 7 |
| $GdGe_9^-$ | 0.37 | [core]$6s^{0.57}4f^{7.00}5d^{1.83}6p^{0.26}$ | 0.02 | 6.98 | 0.28 | 0.01 | 7.29 | 7 |
| $GdGe_{10}^-$ | -0.02 | [core]$6s^{0.43}4f^{7.00}5d^{2.09}6p^{0.51}$ | 0.01 | 6.98 | 0.23 | 0.01 | 7.23 | 7 |
| $GdGe_{11}^-$ | 0.11 | [core]$6s^{0.39}4f^{7.00}5d^{1.85}6p^{0.65}$ | 0.01 | 6.98 | 0.18 | 0.01 | 7.18 | 7 |
| $GdGe_{12}^-$ | -0.26 | [core]$6s^{0.36}4f^{7.00}5d^{2.11}6p^{0.78}$ | 0.00 | 6.98 | 0.17 | 0.01 | 7.16 | 7 |
| $GdGe_{13}^-$ | -0.04 | [core]$6s^{0.46}4f^{7.00}5d^{2.10}6p^{0.49}$ | 0.01 | 6.98 | 0.22 | 0.01 | 7.22 | 7 |
| $GdGe_{14}^-$ | 0.25 | [core]$6s^{0.37}4f^{7.00}5d^{1.82}6p^{0.58}$ | 0.01 | 6.97 | 0.18 | 0.02 | 7.18 | 7 |
| $GdGe_{15}^-$ | -4.10 | [core]$6s^{0.46}4f^{7.00}5d^{4.83}6p^{1.77}$ | 0.00 | 6.97 | 0.19 | 0.01 | 7.17 | 7 |
| $GdGe_{16}^-$ | -4.87 | [core]$6s^{0.47}4f^{7.00}5d^{5.30}6p^{2.05}$ | 0.01 | 6.97 | 0.20 | 0.01 | 7.19 | 7 |
| $GdGe_{17}^-$ | -4.07 | [core]$6s^{0.44}4f^{6.99}5d^{4.84}6p^{1.76}$ | 0.00 | 6.97 | 0.20 | 0.01 | 7.18 | 7 |
| $GdGe_{18}^-$ | -3.17 | [core]$6s^{0.38}4f^{6.99}5d^{4.33}6p^{1.43}$ | 0.00 | 6.97 | 0.19 | 0.01 | 7.17 | 7 |
